# A revised model for Jeffrey nanofluid subject to convective condition and heat generation/absorption

**DOI:** 10.1371/journal.pone.0172518

**Published:** 2017-02-23

**Authors:** Tasawar Hayat, Arsalan Aziz, Taseer Muhammad, Ahmed Alsaedi

**Affiliations:** 1 Department of Mathematics, Quaid-I-Azam University, Islamabad, Pakistan; 2 Nonlinear Analysis and Applied Mathematics (NAAM) Research Group, Department of Mathematics, Faculty of Science, King Abdulaziz University, Jeddah, Saudi Arabia; Tianjin University, CHINA

## Abstract

Here magnetohydrodynamic (MHD) boundary layer flow of Jeffrey nanofluid by a nonlinear stretching surface is addressed. Heat generation/absorption and convective surface condition effects are considered. Novel features of Brownian motion and thermophoresis are present. A non-uniform applied magnetic field is employed. Boundary layer and small magnetic Reynolds number assumptions are employed in the formulation. A newly developed condition with zero nanoparticles mass flux is imposed. The resulting nonlinear systems are solved. Convergence domains are explicitly identified. Graphs are analyzed for the outcome of sundry variables. Further local Nusselt number is computed and discussed. It is observed that the effects of Hartman number on the temperature and concentration distributions are qualitatively similar. Both temperature and concentration distributions are enhanced for larger Hartman number.

## 1. Introduction

The mixture of ultrafine nanometer sized particles and a convectional heat transfer base fluid is known as nanofluid. These nanometer sized particles have different physical and chemical characteristics. Such particles have thermal conductivities remarkably higher than base liquids. The prime use of nanofluids is for thermal conductivity improvement. Nanofluids are significant in various applications including paper and printing, paints and coatings, power generation, drug delivery, cancer therapy, ceramics and food products etc. Further magneto nanofluids are quite prevalent in MHD pumps and accelerators, hyperthermia, cancer tumor treatment, sink float separation, wound treatment and several others. Choi and Eastman [1] proposed the word nanofluid. They concluded that insertion of metallic nanoparticles in the ordinary fluids can dramatically enhance the thermal conductivities and improve the heat transfer performance of these fluids. A model for convective transport in nanofluids was presented by Buongiorno [2]. He pointed out that Brownian diffusion and thermophoresis are the most important slip mechanisms. Boundary layer flow of nanofluid induced by a linear stretching surface was discussed by Khan and Pop [3]. Turkyilmazoglu and Pop [4] examined unsteady natural convection flow of nanofluids by a vertical flat plate with radiation. Double stratification effect in boundary-layer flow of nanofluid by a vertical plate is reported by Ibrahim and Makinde [5]. Further relevant studies involving nanofluids can be seen through the investigations [6–25] and various studies therein.

The study of boundary layer flow caused by a stretchable surface is relevant in numerous industrial and engineering utilizations. Such applications include drawing of copper wires, condensation process, die forging and extrusion of polymer in melt spinning, polymer extrusion, continuous stretching of plastic films, metal extrusion, paper production and fiber production etc. It is noted that stretching of surface is not linear in all the cases. The stretching surface may be nonlinear. Gupta and Gupta [26] declared that the stretching of surface is not linear in plastic process. Vajravelu [27] addressed two-dimensional flow of viscous fluid over a nonlinear stretching surface. Here power law surface velocity distribution *u*_*w*_(*x*) = *cx*^*n*^ is considered. Cortell [28] analyzed heat transfer in the flow past a nonlinear stretching surface. Here two different thermal boundary conditions on the surface namely constant surface temperature and prescribed surface temperature are employed. The boundary layer flow of viscous fluid induced by a nonlinear stretching surface with thermal radiation and viscous dissipation effects is addressed by Cortell [29]. Hydromagnetic flow generated by a nonlinear stretching surface through modified Adomian decomposition method and Pade approximation technique is demonstrated by Hayat et al. [30]. Rana and Bhargava [31] studied flow of nanofluid over a nonlinear stretching surface with heat transfer. Mukhopadhyay [32] addressed the flow and heat transfer characteristics in the flow of nanofluid over a permeable nonlinear stretching surface with partial slip condition. Mustafa et al. [33] explored axisymmetric flow of nanofluid over a nonlinear stretching surface. Magnetohydrodynamic flow of water-based nanofluid bounded by a nonlinear stretching surface with viscous dissipation is analyzed by Mabood et al. [34]. Magnetohydrodynamic flow of second grade nanofluid over a nonlinear stretching surface is reported by Hayat et al. [35].

Recently the non-Newtonian fluids have gained much attention due to their extensive industrial and engineering applications. These applications involve bioengineering and polymeric liquids, plastics manufacturing, food processing, petroleum production, annealing and thinning of copper wires, drawing of stretching sheet through quiescent fluid, aerodynamic extrusion of plastic films etc. The Navier-Stokes equations are not appropriate to characterize the flow of non-Newtonian fluids. A single relation is not sufficient to predict the characteristics of all the non-Newtonian materials. Therefore different types of relations are given in the literature. The fluid model under discussion is called Jeffrey material [36–41]. This model is linear viscoelastic fluid which exhibits the effects of ratio of relaxation to retardation times and retardation time. The Jeffrey fluid is a relatively simpler linear model considering time derivatives while in non-Newtonian fluid mechanics convective derivatives are assumed. Further the analysis of liquid-liquid two-phase flows are widely encountered in several industrial processes such as spray processes, lubrication, natural gas networks, nuclear reactor cooling etc. Thus Gao et al. [42] provided a multivariate weighted complex network analysis to characterize the nonlinear dynamic behavior in two-phase flow. Gao et al. [43] also addressed the multi-frequency complex network to uncover oil-water flows. Slug to churn flow transition with multivariate pseudo Wigner distribution and multivariate multiscale entropy is reported by Gao et al. [44]. Recently Gao et al. [45] provided a four-sector conductance method to explore the low-velocity oil-water two-phase flows.

Present communication explores magnetohydrodynamic (MHD) boundary-layer flow of Jeffrey nanofluid over a nonlinear stretching surface. Jeffrey fluid is assumed to be electrically conducting. We considered the simultaneous effects of heat and mass transfer in the presence of Brownian motion, thermophoresis and heat generation/absorption. Thermal convective [46, 47] and zero nanoparticles mass flux [48, 49] conditions are imposed at the stretching surface. These conditions are studied rarely and more realistic physically. To the best of our knowledge, no such consideration for the flow of Jeffrey nanofluid is made yet. Small magnetic Reynolds number and boundary layer are used in mathematical modelling. The governing nonlinear ordinary differential equations are solved by homotopy analysis method (HAM) [50–60]. This technique for the solutions development has advantages through three directions i.e., (i) It is independent of small/large physical parameters. (ii) It provides a simple way to ensure the convergence of series solutions. (iii) It provides freedom to choose the base functions and related auxiliary linear operators. Temperature and concentration profiles are examined via plots. The local Nusselt number is computed numerically and analyzed.

## 2. Statement

Two-dimensional (2D) flow of Jeffrey nanofluid induced by a surface stretching with nonlinear velocity is considered. Non-uniform magnetic field of strength *B*_0_ acts in the *y*− direction. Small magnetic Reynolds number justifies the absence of induced magnetic field. Non-uniform heat generation/absorption effect is considered. Brownian motion and thermophoresis are present. The *x*− and *y*− axes are along and transverse to the surface respectively. The stretching velocity is *u*_*w*_(*x*) = *ax*^*n*^ (*a*, *n* > 0). The surface temperature is regulated by a convective heating process which is described by heat transfer coefficient *h*_*f*_ and temperature of hot fluid *T*_*f*_ under the surface. Resulting boundary layer problems are

$$\frac{\partial u}{\partial x}+\frac{\partial v}{\partial y}=0,\;$$ (1)

$$u\frac{\partial u}{\partial x}+v\frac{\partial u}{\partial y}=\frac{\nu }{1+\lambda_{1}}\left(\frac{\partial^{2}u}{\partial y^{2}}+\lambda_{2}\left(u\frac{\partial^{3}u}{\partial x\partial y^{2}}-\frac{\partial u}{\partial x}\frac{\partial^{2}u}{\partial y^{2}}+\frac{\partial u}{\partial y}\frac{\partial^{2}u}{\partial x\partial y}+v\frac{\partial^{3}u}{\partial y^{3}}\right)\right)-\frac{\sigma B^{2}(x)}{\rho_{f}}u,\;$$ (2)

$$u\frac{\partial T}{\partial x}+v\frac{\partial T}{\partial y}=\alpha \frac{\partial^{2}T}{\partial y^{2}}+\frac{{\left(\rho c\right)}_{p}}{{\left(\rho c\right)}_{f}}\left(D_{B}\left(\frac{\partial T}{\partial y}\frac{\partial C}{\partial y}\right)+\frac{D_{T}}{T_{\infty }}{\left(\frac{\partial T}{\partial y}\right)}^{2}\right)+\frac{Q(x)}{{\left(\rho c\right)}_{f}}\left(T-T_{\infty }\right),\;$$ (3)

$$u\frac{\partial C}{\partial x}+v\frac{\partial C}{\partial y}=D_{B}\left(\frac{\partial^{2}C}{\partial y^{2}}\right)+\frac{D_{T}}{T_{\infty }}\left(\frac{\partial^{2}T}{\partial y^{2}}\right),$$ (4)

$$u=u_{w}(x)=ax^{n},\;v=0,\;-k\frac{\partial T}{\partial y}=h_{f}\left(T_{f}-T\right),\;D_{B}\frac{\partial C}{\partial y}+\frac{D_{T}}{T_{\infty }}\frac{\partial T}{\partial y}=0\text{ at }y=0,\;$$ (5)

$$u\to 0,\;T\to T_{\infty },\;C\to C_{\infty }\text{ as }y\to \infty .$$ (6)

Note that *u* and *v* depict the flow velocities in the horizontal and vertical directions respectively while *v* (= *μ* / *ρ*_*f*_), *μ* and *ρ*_*f*_ show the kinematic viscosity, dynamic viscosity and density of base liquid respectively. The ratio of relaxation to retardation times and the retardation time are represented by *λ*_1_ and *λ*_2_. Here *σ* represents the electrical conductivity, $B(x)=B_{0}x^{\frac{n-1}{2}}$ the non-uniform magnetic field, *T* the temperature, *α* = *k* / (*ρc*)_*f*_, *k*, (*ρc*)_*f*_ and (*ρc*)_*p*_ the thermal diffusivity, thermal conductivity, heat capacity of liquid and effective heat capacity of nanoparticles respectively, *Q*(*x*) = *Q*_0_*x*^*n*−1^ the non-uniform heat generation/absorption coefficient, *D*_*B*_ the Brownian diffusivity, *C* the concentration, *D*_*T*_ the thermophoretic diffusion coefficient, *a* the positive constant and *T*_∞_ and *C*_∞_ the ambient fluid temperature and concentration respectively. Putting

$$\begin{array}{c} u=ax^{n}f^{\prime }(\zeta ),\;v=-{\left(\frac{a\nu (n+1)}{2}\right)}^{1/2}{\left(x\right)}^{\frac{n-1}{2}}\{f(\zeta )+\frac{n-1}{n+1}\zeta f^{\prime }(\zeta )\},\\ \theta (\zeta )=\frac{T-T_{\infty }}{T_{f}-T_{\infty }},\;\phi (\zeta )=\frac{C-C_{\infty }}{C_{\infty }},\;\zeta ={\left(\frac{a(n+1)}{2\nu }\right)}^{1/2}{\left(x\right)}^{\frac{n-1}{2}}y, \end{array}\}\;$$ (7)

Eq (1) is trivially satisfied while Eqs (2)–(6) are reduced to

  f‴+β1((3n−12) (f″)2−(n+12) ff′′′′+(n−1) ff‴)   +(1+λ1) (ff″−(2nn+1)(f′)2−(2n+1)(Ha)2f′)=0,  (8)

$$\theta^{\prime \prime }+\text{Pr}(f\theta^{\prime }+N_{b}\theta^{\prime }\phi \text{'}+N_{t}\theta^{\prime^{2}}+\left(\frac{2}{n+1}\right)\;S_{1}\theta )=0,\;$$ (9)

$$\phi "+Le\text{Pr}f\phi \prime +\frac{N_{t}}{N_{b}}\theta^{\prime \prime }=0,\;$$ (10)

$$f=0,\;f^{\prime }=1,\;\theta^{\prime }=-\gamma \left(1-\theta \left(0\right)\right),\;N_{b}\phi \prime +N_{t}\theta^{\prime }=0\text{ at }\zeta =0,\;$$ (11)

$$f^{\prime }\to 0,\;\theta \to 0,\;\phi \to 0\text{ when }\zeta \to \infty .$$ (12)

Here *β*_1_ denotes local Deborah number, *Ha* Hartman number, *γ* Biot number, *S*_1_ heat generation/absorption parameter, Pr Prandtl number, *N*_*b*_ Brownian motion parameter, *N*_*t*_ thermophoresis parameter and *Le* Lewis number. The definitions of these parameters are

$$\begin{array}{c} \beta_{1}=\lambda_{2}ax^{n-1},\;{\left(Ha\right)}^{2}=\frac{\sigma B_{0}^{2}}{a\rho_{f}},\;\gamma =\frac{h_{f}}{k}\sqrt{\frac{\nu }{a}},\;S_{1}=\frac{Q_{0}}{a{\left(\rho c\right)}_{f}}\\ N_{b}=\frac{{\left(\rho c\right)}_{p}D_{B}C_{\infty }}{{\left(\rho c\right)}_{f}\nu },\;N_{t}=\frac{{\left(\rho c\right)}_{p}D_{T}\left(T_{f}-T_{\infty }\right)}{{\left(\rho c\right)}_{f}\nu T_{\infty }},\;Le=\frac{\alpha }{D_{B}},\text{ Pr}=\frac{\nu }{\alpha }. \end{array}\}\;$$ (13)

Local Nusselt number is given by

$$Re_{x}^{-1/2}Nu_{x}=-{\left(\frac{n+1}{2}\right)}^{1/2}\theta^{\prime }(0).$$ (14)

The non-dimensional local Sherwood number is identically zero and Re_*x*_ = *u*_*w*_*x/v* represents the local Reynolds number.

## 3. Solutions by HAM

The appropriate initial approximations and auxiliary linear operators for approximate series solutions by homotopy analysis method (HAM) are

$$f_{0}(\zeta )=1-e^{-\zeta },\;\theta_{0}(\zeta )=\frac{\gamma }{1+\gamma }e^{-\zeta },\;\phi_{0}(\zeta )=-\frac{\gamma }{1+\gamma }\frac{N_{t}}{N_{b}}e^{-\zeta },\;$$ (15)

$$L_{f}=\frac{d^{3}f}{d\zeta^{3}}-\frac{df}{d\zeta },\;L_{\theta }=\frac{d^{2}\theta }{d\zeta^{2}}-\theta ,\;L_{\phi }=\frac{d^{2}\phi }{d\zeta^{2}}-\phi ,$$ (16)

subject to

$$L_{f}\left[B_{1}^{*}+B_{2}^{*}e^{\zeta }+B_{3}^{*}e^{-\zeta }\right]=0,\;L_{\theta }\left[B_{4}^{*}e^{\zeta }+B_{5}^{*}e^{-\zeta }\right]=0,\;L_{\phi }\left[B_{6}^{*}e^{\zeta }+B_{7}^{*}e^{-\zeta }\right]=0,\;$$ (17)

where $B_{j}^{*}$ (*j* = 1–7) denote the arbitrary constants. Deformation problems at zeroth-order are

$$(1-þ)L_{f}\left[\overset{⌣}{f}(\zeta ,\;þ)-f_{0}(\zeta )\right]=þ\hbar_{f}N_{f}[\overset{⌣}{f}(\zeta ,\;þ)],\;$$ (18)

$$(1-þ)L_{\theta }\left[\overset{⌣}{\theta }(\zeta ,\;þ)-\theta_{0}(\zeta )\right]=þ\hbar_{\theta }N_{\theta }[\overset{⌣}{f}(\zeta ,\;þ),\;\overset{⌣}{\theta }(\zeta ,\;þ),\;\overset{⌣}{\phi }(\zeta ,\;þ)],\;$$ (19)

$$(1-þ)L_{\phi }\left[\overset{⌣}{\phi }(\zeta ,\;þ)-\phi_{0}(\zeta )\right]=þ\hbar_{\phi }N_{\phi }[\overset{⌣}{f}(\zeta ,\;þ),\;\overset{⌣}{\theta }(\zeta ,\;þ),\;\overset{⌣}{\phi }(\zeta ,\;þ)],\;$$ (20)

$$\begin{array}{c} \overset{⌣}{f}(0,\;þ)=0,\;{\overset{⌣}{f}}^{\prime }(0,\;þ)=1,\;{\overset{⌣}{f}}^{\prime }(\infty ,\;þ)=0,\;{\overset{⌣}{\theta }}^{\prime }(0,\;þ)=-\gamma \left(1-\overset{⌣}{\theta }(0,\;þ)\right),\\ \overset{⌣}{\theta }(\infty ,\;þ)=0,\;Nb{\overset{⌣}{\phi }}^{\prime }(0,\;þ)+Nt{\overset{⌣}{\theta }}^{\prime }(0,\;þ)=0,\;\overset{⌣}{\phi }(\infty ,\;þ)=0, \end{array}$$ (21)

Nf[f⌣(ζ; þ)]=∂3f⌣∂ζ3+β1((3n−12) (∂2f⌣∂ζ2)2−(n+12) f⌣∂4f⌣∂ζ4+(n−1) f⌣∂3f⌣∂ζ3) +(1+λ1) (f⌣∂2f⌣∂ζ2−(2nn+1) (∂f⌣∂ζ)2−(2n+1)(Ha)2∂f⌣∂ζ),  (22)

$$N_{\theta }\left[\overset{⌣}{f}(\zeta ;\;þ),\;\overset{⌣}{\theta }(\zeta ,\;þ),\;\overset{⌣}{\phi }(\zeta ,\;þ)\right]=\frac{1}{\text{Pr}}\frac{\partial^{2}\overset{⌣}{\theta }}{\partial \zeta^{2}}+\overset{⌣}{f}\frac{\partial \overset{⌣}{\theta }}{\partial \zeta }+N_{b}\frac{\partial \overset{⌣}{\theta }}{\partial \zeta }\frac{\partial \overset{⌣}{\theta }}{\partial \zeta }+N_{t}{\left(\frac{\partial \overset{⌣}{\theta }}{\partial \zeta }\right)}^{2}+\left(\frac{2}{n+1}\right)\;S_{1}\overset{⌣}{\theta },\;$$ (23)

$$N_{\phi }\left[\overset{⌣}{f}(\zeta ;\;þ),\;\overset{⌣}{\theta }(\zeta ,\;þ),\;\overset{⌣}{\phi }(\zeta ,\;þ)\right]=\frac{\partial^{2}\overset{⌣}{\phi }}{\partial \zeta^{2}}+Le\text{Pr}\overset{⌣}{f}\frac{\partial \overset{⌣}{\phi }}{\partial \zeta }+\frac{N_{t}}{N_{b}}\frac{\partial^{2}\overset{⌣}{\theta }}{\partial \zeta^{2}}.$$ (24)

Here þ ∈[0,1] represents the embedding parameter, ℏ_*f*_, ℏ_*θ*_ and ℏ_*ϕ*_ the non-zero auxiliary parameters and **N**_*f*_, **N**_*θ*_ and **N***ϕ* the nonlinear operators. For þ = 0 and þ = 1 we have

$$\overset{⌣}{f}(\zeta ;\;0)=f_{0}(\zeta ),\;\overset{⌣}{f}(\zeta ;\;1)=f(\zeta ),\;$$ (25)

$$\overset{⌣}{\theta }(\zeta ,\;0)=\theta_{0}(\zeta ),\;\overset{⌣}{\theta }(\zeta ,\;1)=\theta (\zeta ),\;$$ (26)

$$\overset{⌣}{\phi }(\zeta ,\;0)=\phi_{0}(\zeta ),\;\overset{⌣}{\phi }(\zeta ,\;1)=\phi (\zeta ).$$ (27)

When þ changes from 0 to 1 then $\overset{⌣}{f}(\zeta ;þ)$, $\overset{⌣}{\theta }(\zeta ,þ)$ and $\overset{⌣}{\phi }(\zeta ,þ)$ vary from primary approximations *f*_0_(*ζ*), *θ*_0_(*ζ*) and *ϕ*_0_(*ζ*) to the desired solutions *f*(*ζ*), *θ*(*ζ*) and *ϕ*(*ζ*). The following expressions by Taylor's series expansion can be written as

$$\overset{⌣}{f}(\zeta ;\;þ)=f_{0}(\zeta )+\overset{\infty }{\underset{\hat{m}=1}{\sum }}f_{\hat{m}}(\zeta ){þ}^{\hat{m}},\;f_{\hat{m}}(\zeta )={\frac{1}{\hat{m}!}\frac{\partial^{\hat{m}}\overset{⌣}{f}(\zeta ,\;þ)}{\partial {þ}^{\hat{m}}}|}_{þ=0},\;$$ (28)

$$\overset{⌣}{\theta }(\zeta ,\;þ)=\theta_{0}(\zeta )+\overset{\infty }{\underset{\hat{m}=1}{\sum }}\theta_{\hat{m}}(\zeta ){þ}^{\hat{m}},\;\theta_{\hat{m}}(\zeta )={\frac{1}{\hat{m}!}\frac{\partial^{\hat{m}}\overset{⌣}{\theta }(\zeta ,\;þ)}{\partial {þ}^{\hat{m}}}|}_{þ=0},\;$$ (29)

$$\overset{⌣}{\phi }(\zeta ,\;þ)=\phi_{0}(\zeta )+\overset{\infty }{\underset{\hat{m}=1}{\sum }}\phi_{\hat{m}}(\zeta ){þ}^{\hat{m}},\;\phi_{\hat{m}}(\zeta )={\frac{1}{\hat{m}!}\frac{\partial^{\hat{m}}\overset{⌣}{\phi }(\zeta ,\;þ)}{\partial {þ}^{\hat{m}}}|}_{þ=0}.\;$$ (30)

The convergence of above series expressions strongly depends upon ℏ_*f*,_ ℏ_*θ*_ and ℏ_*ϕ*_. The values of ℏ_*f*,_ ℏ_*θ*_ and ℏ_*ϕ*_ are chosen so that Eqs (28)–(30) converge at þ = 1 then

$$f(\zeta )=f_{0}(\zeta )+\overset{\infty }{\underset{\hat{m}=1}{\sum }}f_{\hat{m}}(\zeta ),\;$$ (31)

$$\theta (\zeta )=\theta_{0}(\zeta )+\overset{\infty }{\underset{\hat{m}=1}{\sum }}\theta_{\hat{m}}(\zeta ),\;$$ (32)

$$\phi (\zeta )=\phi_{0}(\zeta )+\overset{\infty }{\underset{\hat{m}=1}{\sum }}\phi_{\hat{m}}(\zeta ).$$ (33)

The $\hat{m}$ th-order deformation problems can be expressed as follows:

$$L_{f}\left[f_{\hat{m}}(\zeta )-\chi_{\hat{m}}f_{\hat{m}-1}(\zeta )\right]=\hbar_{f}{\tilde{R}}_{f}^{\hat{m}}(\zeta ),\;$$ (34)

$$L_{\theta }\left[\theta_{\hat{m}}(\zeta )-\chi_{\hat{m}}\theta_{\hat{m}-1}(\zeta )\right]=\hbar_{\theta }{\tilde{R}}_{\theta }^{\hat{m}}(\zeta ),\;$$ (35)

$$L_{\phi }\left[\phi_{\hat{m}}(\zeta )-\chi_{\hat{m}}\phi_{\hat{m}-1}(\zeta )\right]=\hbar_{\phi }{\tilde{R}}_{\phi }^{\hat{m}}(\zeta ),\;$$ (36)

$$\begin{array}{c} f_{\hat{m}}(0)=f_{\hat{m}}^{\prime }(0)=f_{\hat{m}}^{\prime }(\infty )=0,\;\theta_{\hat{m}}^{\prime }(0)-\gamma \theta_{\hat{m}}(0)=0,\\ \;Nb\phi_{\hat{m}}^{{}^{\prime }}(0)+Nt\theta_{\hat{m}}^{{}^{\prime }}(0)=0,\;\theta_{\hat{m}}(\infty )=\phi_{\hat{m}}(\infty )=0, \end{array}\}\;$$ (37)

$$\begin{array}{c} {\tilde{R}}_{f}^{\hat{m}}(\zeta )=f_{\hat{m}-1}^{\prime \prime \prime }+\beta_{1}\sum_{\hat{m}-1}^{k=0}\left(\left(\frac{3n-1}{2}\right)\;f_{\hat{m}-1-k}^{\prime \prime }f_{k}^{\prime \prime }-\left(\frac{n+1}{2}\right)\;f_{\hat{m}-1-k}f_{k}^{\prime \prime \prime \prime }+\left(n-1\right)\;f_{\hat{m}-1-k}f_{k}^{\prime \prime \prime }\right)\;\\ +(1+\lambda_{1})\sum_{\hat{m}-1}^{k=0}\left(f_{\hat{m}-1-k}f_{k}^{\prime \prime }-\left(\frac{2n}{n+1}\right)\;f_{\hat{m}-1-k}^{\prime }f_{k}^{\prime }\right)\;-\left(1+\lambda_{1}\right)\;\left(\frac{2}{n+1}\right){\left(Ha\right)}^{2}f_{\hat{m}-1}^{\prime },\; \end{array}$$ (38)

$$\begin{array}{c} {\tilde{R}}_{\theta }^{\hat{m}}(\zeta )=\frac{1}{\text{Pr}}\theta_{\hat{m}-1}^{\prime \prime }+\sum_{k=0}^{\hat{m}-1}f_{\hat{m}-1-k}\theta_{k}^{\prime }+N_{b}\sum_{k=0}^{\hat{m}-1}\theta_{\hat{m}-1-k}^{{}^{\prime }}\phi_{k}^{\prime }\\ +N_{t}\sum_{k=0}^{\hat{m}-1}\theta_{\hat{m}-1-k}^{{}^{\prime }}\theta_{k}^{\prime }+\left(\frac{2}{n+1}\right)\;S_{1}\theta_{\hat{m}-1},\; \end{array}$$ (39)

$${\tilde{R}}_{\phi }^{\hat{m}}(\zeta )=\phi_{\hat{m}-1}^{\prime \prime }+Le\text{Pr}\overset{\hat{m}-1}{\underset{k=0}{\sum }}\left(f_{\hat{m}-1-k}\phi_{k}^{\prime }\right)+\frac{N_{t}}{N_{b}}\theta_{\hat{m}-1}^{\prime \prime },\;$$ (40)

$$\chi_{\hat{m}}=\{\begin{array}{c} 0,\;\hat{m}\leq 1,\\ 1,\;\hat{m}>1. \end{array}\;$$ (41)

General expressions of $\left(f_{\hat{m}},\theta_{\hat{m}},\phi_{\hat{m}}\right)$ through special solutions $\left(f_{\hat{m}}^{*},\;\theta_{\hat{m}}^{*},\;\phi_{\hat{m}}^{*}\right)$ are presented by the following expressions:

$$f_{\hat{m}}(\zeta )=f_{\hat{m}}^{*}(\zeta )+B_{1}^{*}+B_{2}^{*}e^{\zeta }+B_{3}^{*}e^{-\zeta },\;$$ (42)

$$\theta_{\hat{m}}(\zeta )=\theta_{\hat{m}}^{*}(\zeta )+B_{4}^{*}e^{\zeta }+B_{5}^{*}e^{-\zeta },\;$$ (43)

$$\phi_{\hat{m}}(\zeta )=\phi_{\hat{m}}^{*}(\zeta )+B_{6}^{*}e^{\zeta }+B_{7}^{*}e^{-\zeta },$$ (44)

in which the constants $B_{j}^{*}$ (*j* = 1–7) through the boundary condition (37) are given by

$$B_{2}^{*}=B_{4}^{*}=B_{6}^{*}=0,\;B_{3}^{*}={\frac{\partial f_{\hat{m}}^{*}(\zeta )}{\partial \zeta }|}_{\zeta =0},\;B_{1}^{*}=-B_{3}^{*}-f_{\hat{m}}^{*}(0),\;$$ (45)

$$B_{5}^{*}=\frac{1}{1+\gamma }\left({\frac{\partial \theta_{\hat{m}}^{*}(\zeta )}{\partial \zeta }|}_{\zeta =0}-\gamma \theta_{\hat{m}}^{*}(0)\right),\;$$ (46)

$$B_{7}^{*}={\frac{\partial \phi_{\hat{m}}^{*}(\zeta )}{\partial \zeta }|}_{\zeta =0}+\frac{N_{t}}{N_{b}}\left(-B_{5}^{*}+{\frac{\partial \theta_{\hat{m}}^{*}(\zeta )}{\partial \zeta }|}_{\zeta =0}\right).$$ (47)

## 4. Convergence analysis

**Fig 1:**
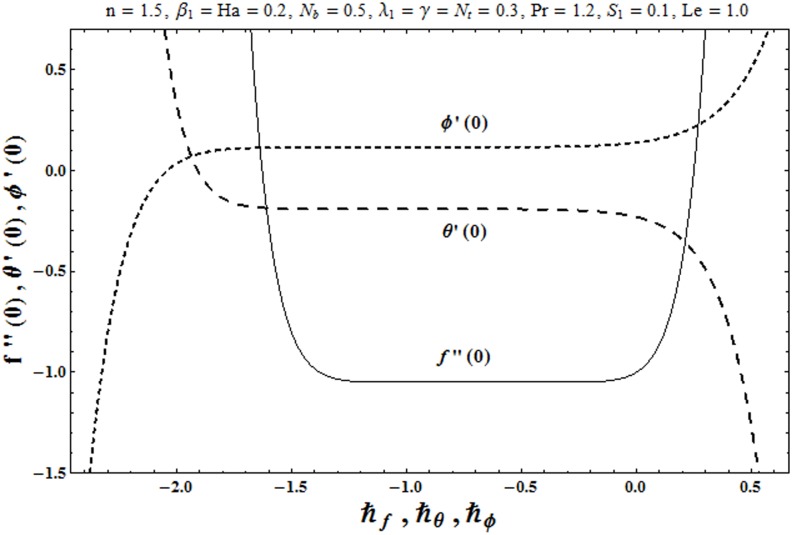
The ℏ − curves for *f*, *θ* and *ϕ*.

The expressions (31)–(33) contain ℏ_*f*,_ ℏ_*θ*_ and ℏ_*ϕ*_. Obviously the convergence is accelerated by the auxiliary parameters ℏ_*f*,_ ℏ_*θ*_ and ℏ_*ϕ*_ for the series solutions. For appropriate values of ℏ_*f*,_ ℏ_*θ*_ and ℏ_*ϕ*_, the ℏ− curves at 15th order of approximations are sketched. It is apparent from Fig 1 that the admissible ranges of ℏ_*f*,_ ℏ_*θ*_ and ℏ_*ϕ*_ are −1.35 ≤ ℏ_*f*_ ≤ −0.15, −1.50 ≤ ℏ_*θ*_ ≤ −0.15 and −1.60 ≤ ℏ_*ϕ*_ ≤ −0.15 respectively. The residual errors for velocity, temperature and concentration distributions are calculated through the following expressions:

$$\Delta_{m}^{f}=\int_{0}^{1}{\left[{\tilde{R}}_{\hat{m}}^{f}\left(\zeta ,\hbar_{f}\right)\right]}^{2}d\zeta ,\;$$ (48)

$$\Delta_{m}^{\theta }=\int_{0}^{1}{\left[{\tilde{R}}_{\hat{m}}^{\theta }\left(\zeta ,\hbar_{\theta }\right)\right]}^{2}d\zeta ,\;$$ (49)

$$\Delta_{m}^{\phi }=\int_{0}^{1}{\left[{\tilde{R}}_{\hat{m}}^{\phi }\left(\zeta ,\hbar_{\phi }\right)\right]}^{2}d\zeta .$$ (50)

**Fig 2:**
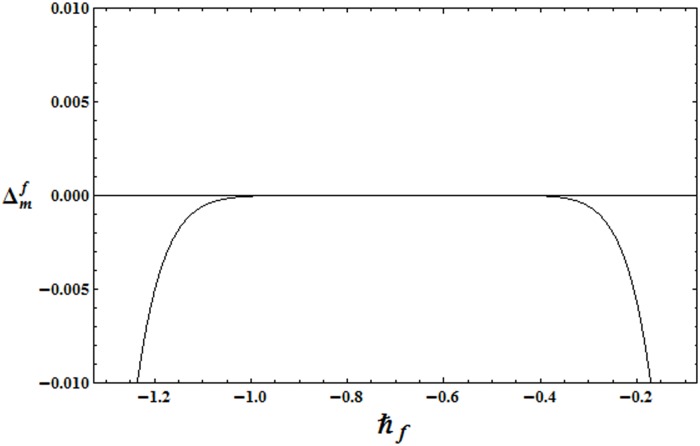
ℏ_*f*_ − curve for the residual error $\Delta_{m}^{f}$.

**Fig 3:**
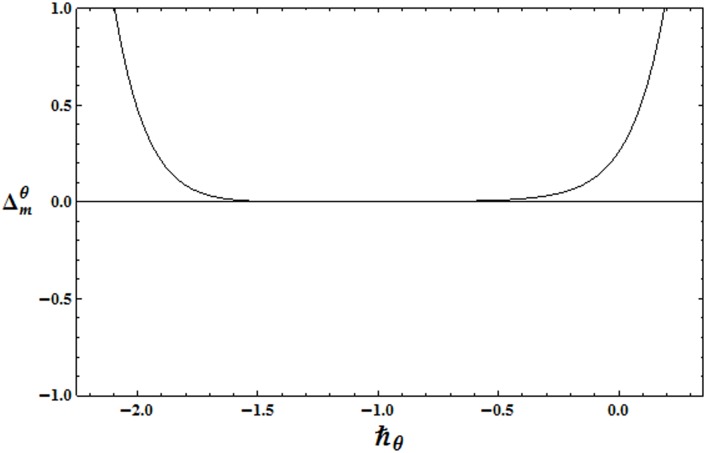
ℏ_*f*_ − curve for the residual error $\Delta_{m}^{\theta }$.

**Fig 4:**
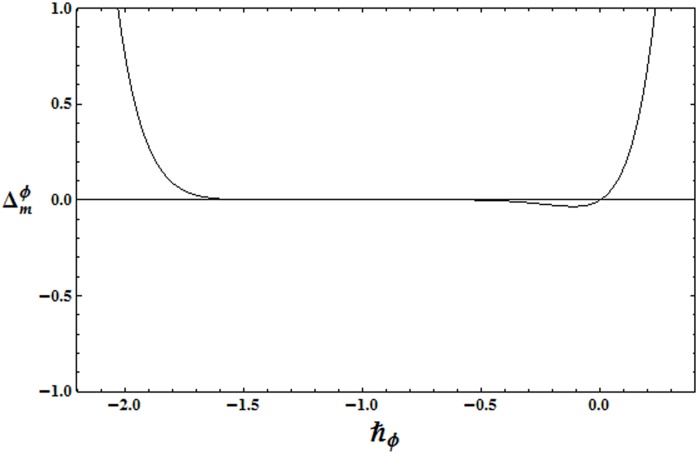
ℏ_*ϕ*_ − curve for the residual error $\Delta_{m}^{\phi }$.

To get the suitable range for ℏ, the ℏ− curves for the residual errors of velocity, temperature and concentration distributions are plotted in the Figs 2–4. It is observed that the correct results up to fifth decimal place are obtained for values of ℏ from this range. Table 1 presents that the 24th order of deformations is enough for the convergent series solutions of velocity, temperature and concentration distributions.

**Table 1:** Homotopic solutions convergence when *n* = 1.5, *β*_1_ = *Ha* = 0.2, *N*_*b*_ = 0.5, *λ*_1_ = *γ* = *N*_*t*_ = 0.3, Pr = 1.2, S1 = 0.1, and *Le* = 1.0.

Order of approximations	−*f*′′(0)	−*θ*′(0)	−*ϕ*′(0)
1	1.05680	0.21543	0.12926
8	1.04981	0.19237	0.11542
15	1.04981	0.19085	0.11451
24	1.04981	0.19065	0.11439
30	1.04981	0.19065	0.11439
40	1.04981	0.19065	0.11439
50	1.04981	0.19065	0.11439

## 5. Discussion

**Fig 5:**
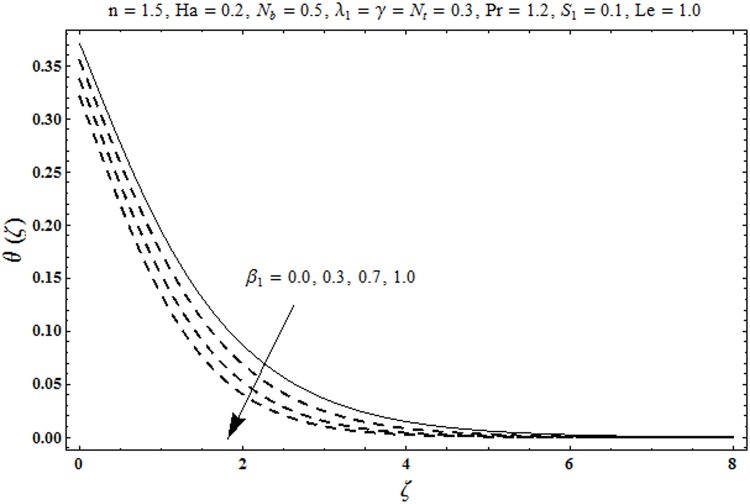
Plots of temperature *θ*(*ζ*) for local Deborah number *β*_1_.

**Fig 6:**
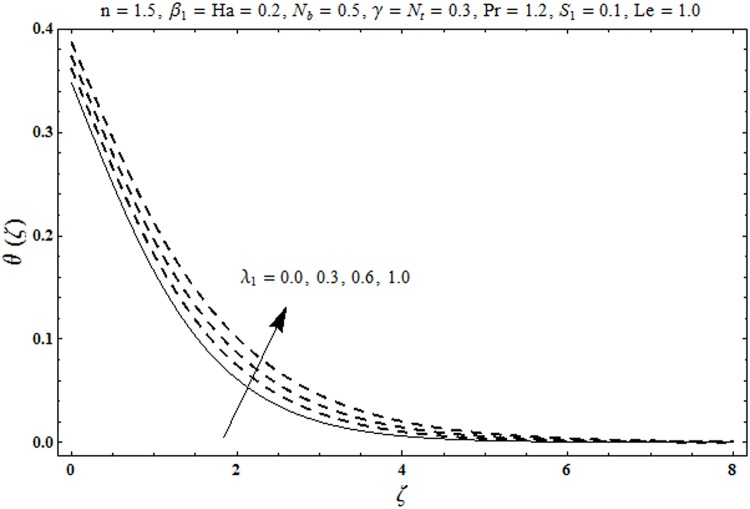
Plots of temperature profile *θ*(*ζ*) for ratio of relaxation to retardation time *λ*_1_.

**Fig 7:**
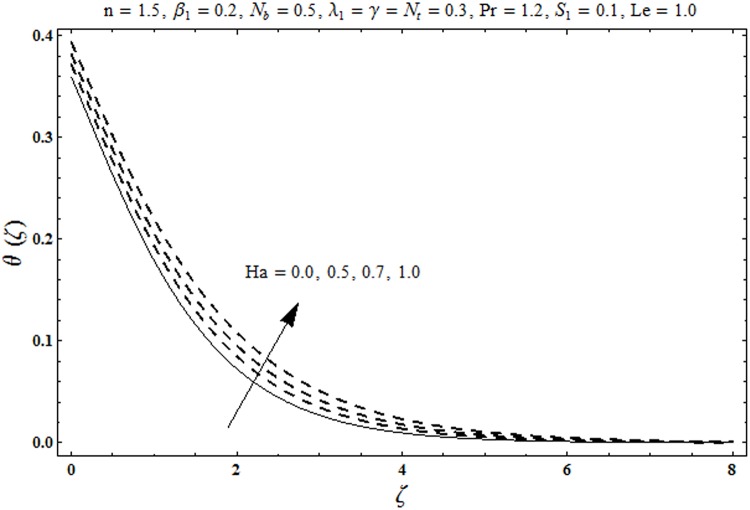
Plots of temperature profile *θ(ζ)* for Hartman number *Ha*.

**Fig 8:**
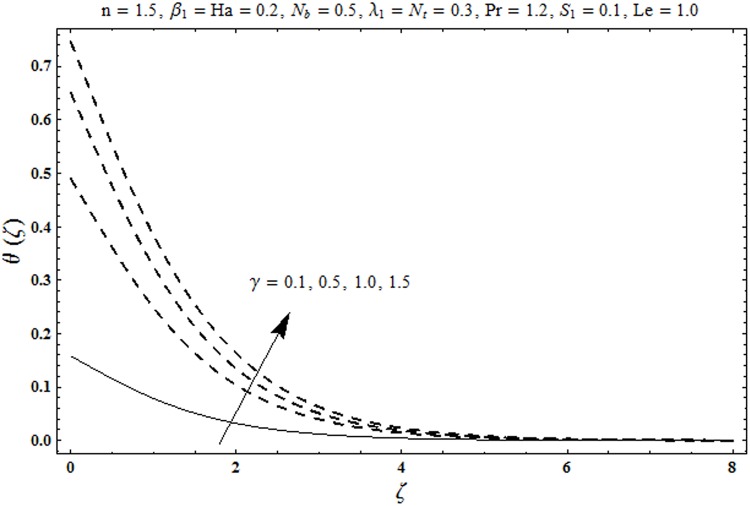
Plots of temperature profile *θ(ζ)* for Biot number *γ*.

**Fig 9:**
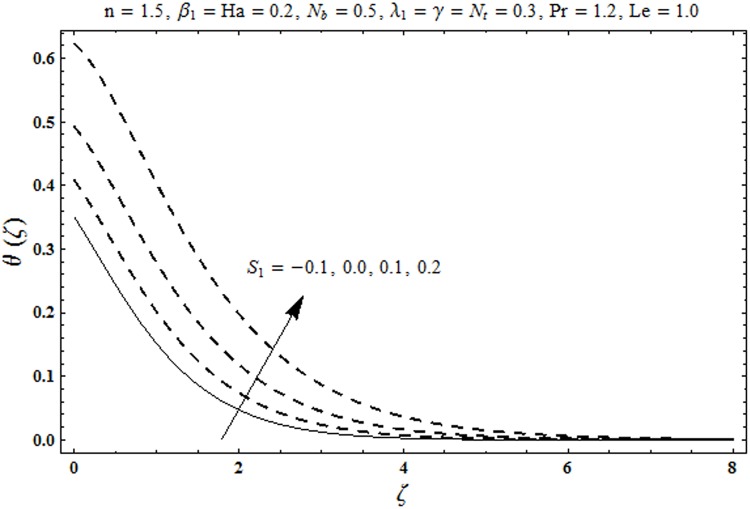
Plots of temperature profile *θ(ζ)* for heat generation/absorption parameter *S*_1_.

**Fig 10:**
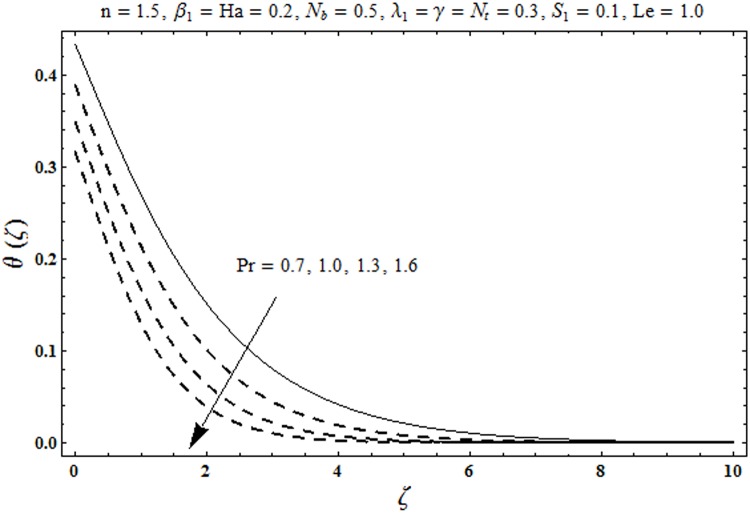
Plots of temperature profile *θ(ζ)* for Prandtk number Pr.

**Fig 11:**
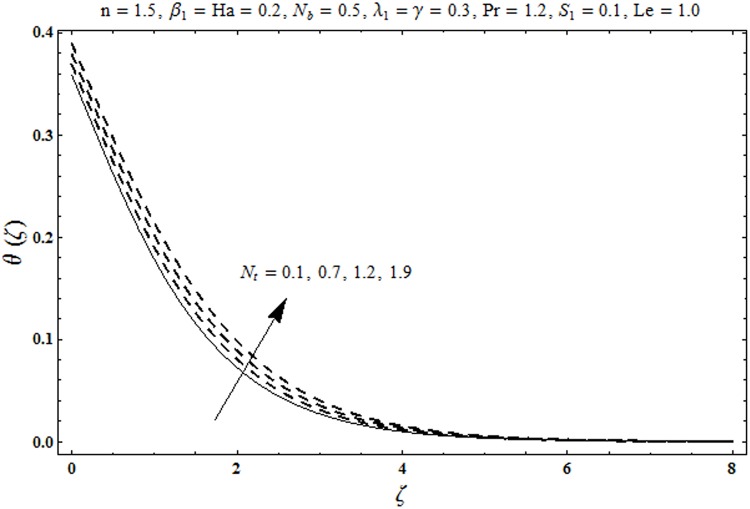
Plots of temperature profile *θ(ζ)* for thermophoresis parameter *N*_*t*._

**Fig 12:**
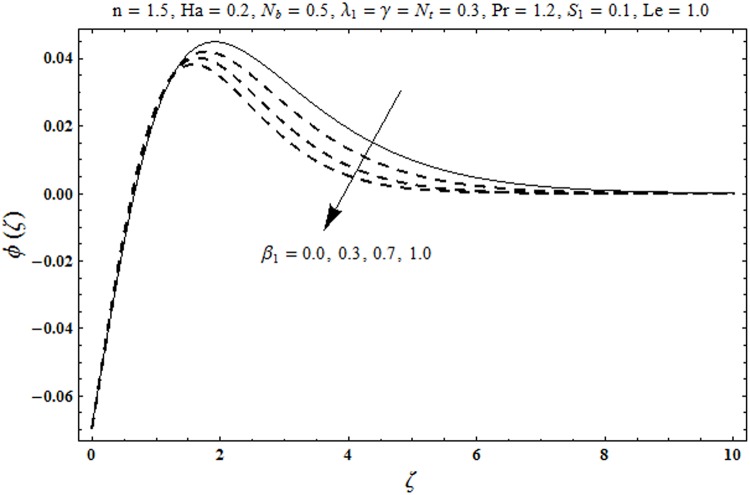
Plots of concentration profile *ϕ*(*ζ*) for local Deborah number *β*_1_.

**Fig 13:**
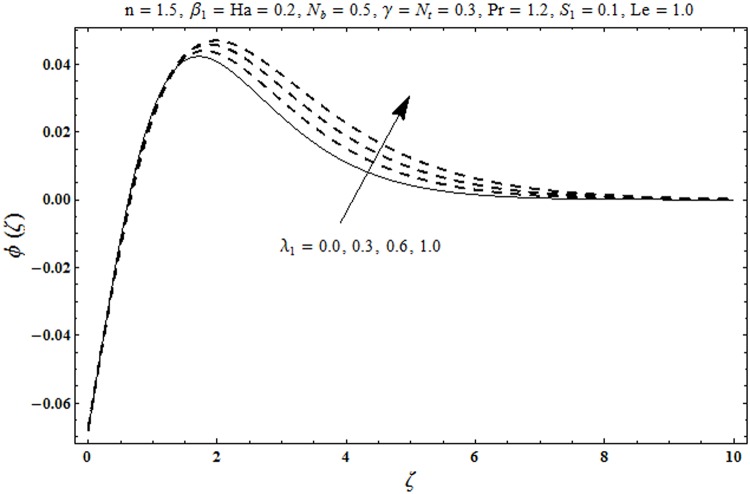
Plots of concentration profile *ϕ*(*ζ*) for ratio of relaxation to retardation time *λ*_1_.

**Fig 14:**
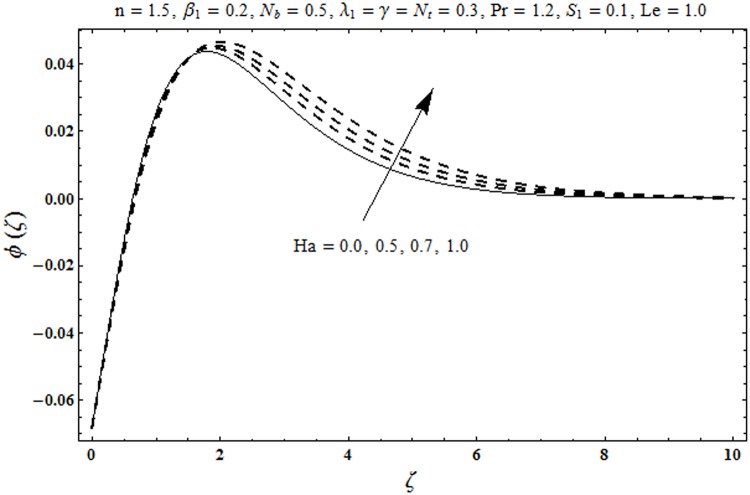
Plots of concentration profile *ϕ*(*ζ*) for Hartman number *Ha*.

**Fig 15:**
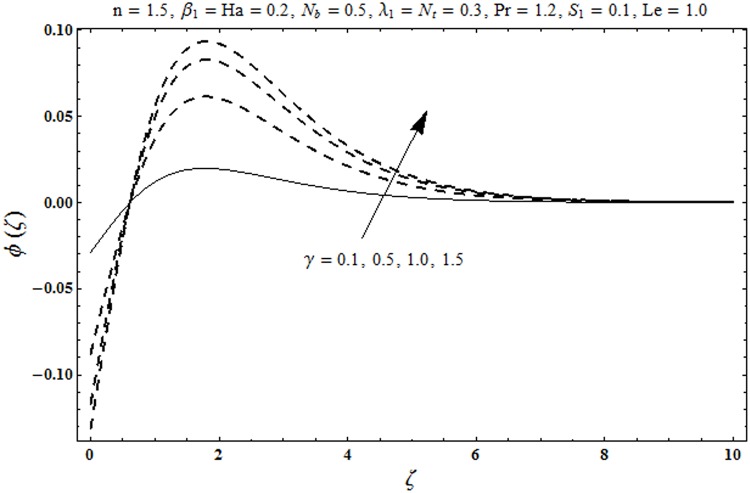
Plots of concentration profile *ϕ*(*ζ*) for Boit number γ.

**Fig 16:**
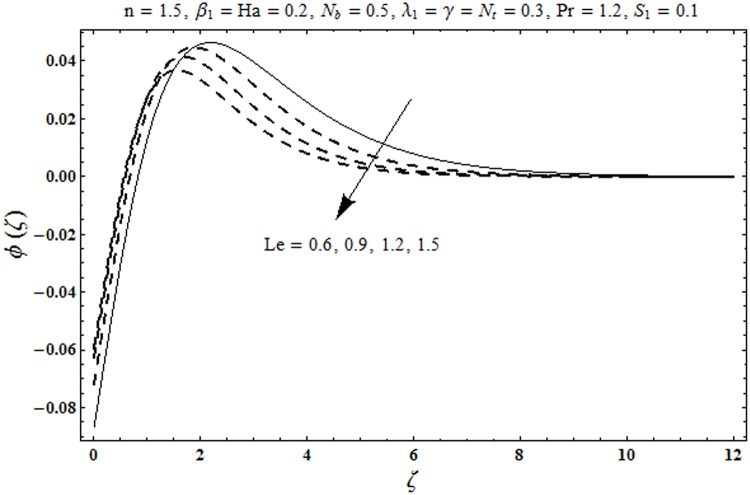
Plots of concentration profile *ϕ*(*ζ*) for Lewis number *Le*.

**Fig 17:**
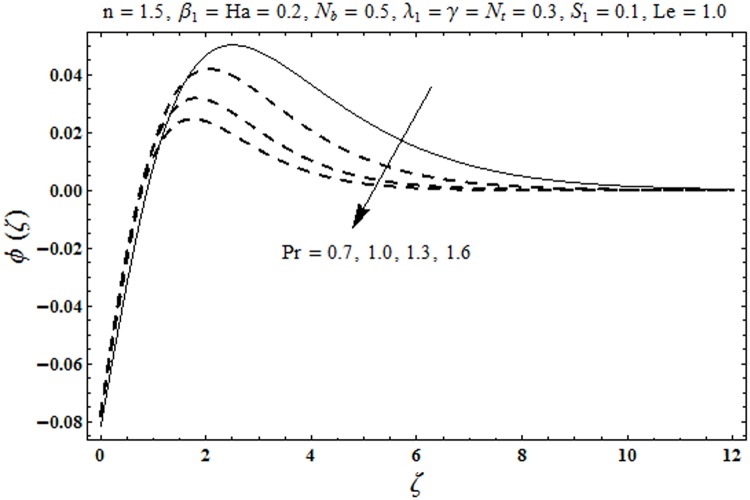
Plots of concentration profile *ϕ*(*ζ*) for Prandtl number Pr.

**Fig 18:**
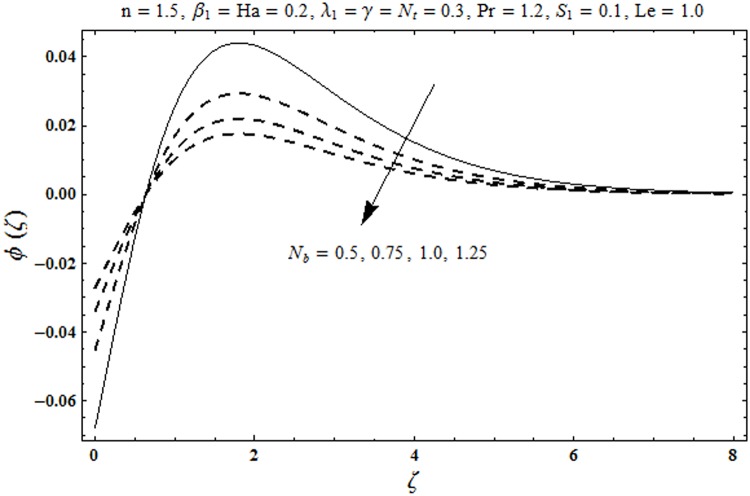
Plots of concentration profile *ϕ*(*ζ*) for Brownian motion parameter *N*_*b*_.

**Fig 19:**
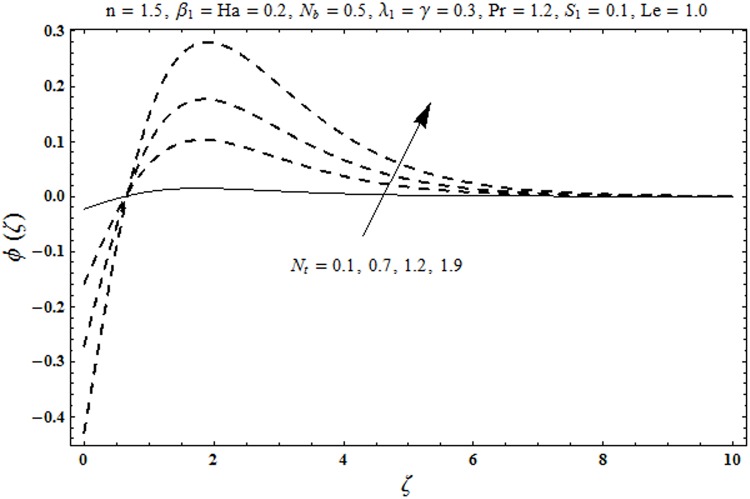
Plots of concentration profile *ϕ*(*ζ*) for thermophoresis motion parameter *N*_*t*_.

This portion organized the impacts of local Deborah number *β*_1_, Hartman number *Ha*, Brownian motion parameter *N*_*b*_, ratio of relaxation to retardation times *λ*_1_, Biot number *γ*, thermophoresis parameter *N*_*t*_, Prandtl number Pr, heat generation/absorption parameter *S*_1_ and Lewis number *Le* on the temperature *θ*(*ζ*) and concentration *ϕ*(*ζ*). Fig 5 illustrates that how local Deborah number *β*_1_ affects the temperature distribution *θ*(*ζ*). It is analyzed that temperature *θ*(*ζ*) and related thermal layer thickness are decreased for larger local Deborah number *β*_1_. Physically there exists a direct relationship between local Deborah number *β*_1_ and retardation time. Hence by increasing local Deborah number *β*_1_, the retardation time is also enhanced. Such enhancement in retardation time corresponds to lower temperature distribution *θ*(*ζ*) and thinner thermal layer thickness. Influence of *λ*_1_ on temperature distribution *θ*(*ζ*) is shown in Fig 6. For larger *λ*_1,_ the relaxation time increases and retardation time decays. Thus temperature distribution *θ*(*ζ*) and thermal layer thickness are increased. Fig 7 presents variation in temperature distribution *θ*(*ζ*) for Hartman number *Ha*. An increase in Hartman number corresponds to more temperature *θ*(*ζ*) and thermal layer thickness. As expected the magnetic field introduces the retarding body force that acts transverse to the direction of an applied magnetic field. It retards the fluid motion and as a result the temperature distribution *θ*(*ζ*) enhances. This body force is known as Lorentz force. Fig 8 presents the impact of Biot number *γ* on temperature *θ*(*ζ*). Stronger convection is caused by increasing Biot number *γ*. Therefore the temperature *θ*(*ζ*) and thermal layer thickness are enhanced. Variation in temperature *θ*(*ζ*) due to heat generation/absorption parameter *S*_1_ is shown in Fig 9. Here *S*_1_ > 0 represents heat generation and *S*_1_ < 0 yields heat absorption. Temperature profile and related thermal layer thickness have increasing behavior for heat generation but it is not the case for heat absorption. Fig 10 demonstrates the variation of temperature *θ*(*ζ*) for Prandtl number Pr. It is observed that temperature *θ*(*ζ*) and thermal layer thickness are decreasing functions of Pr. Physically Prandtl number Pr is an integral part of thermal diffusivity. Thermal diffusivity is responsible for lower temperature *θ*(*ζ*) and thermal layer thickness. Higher values of Prandtl number yields weaker thermal diffusivity which corresponds to lower temperature and less thickness of thermal layer. Fig 11 is drawn for impact of thermophoresis parameter *N*_*t*_ on temperature *θ*(*ζ*). Larger thermophoresis parameter *N*_*t*_ lead to higher temperature and more thermal layer thickness. Actually an enhancement in *N*_*t*_ yields a stronger thermophoretic force which allows deeper migration of nanoparticles in the fluid. Far away from the surface there is higher temperature field and more thickness of thermal layer. Fig 12 is sketched to examine concentration field *ϕ*(*ζ*) for local Deborah number *β*_1_. Here concentration field is weaker for larger values of *β*_1_. Concentration field *ϕ*(*ζ*) enhances when *λ*_1_ increases (see Fig 13). Fig 14 shows impact of Hartman number *Ha* on concentration *ϕ*(*ζ*). The concentration *ϕ*(*ζ*) and associated layer thickness are enhanced for larger Hartman number. From Fig 15 we observed that an increase in Biot number *γ* yields an enhancement in concentration profile *ϕ*(*ζ*) and its related boundary layer thickness. Larger Lewis number *Le* indicate decay in the concentration field *ϕ*(*ζ*) (see Fig 16). Physically Lewis number is based on Brownian diffusivity. An increase in Lewis number *Le* yields weaker Brownian diffusivity. Such weaker Brownian diffusivity corresponds to lower concentration field *ϕ*(*ζ*). Fig 17 addresses variation of Prandtl number Pr on concentration *ϕ*(*ζ*). The concentration *ϕ*(*ζ*) and associated thickness of boundary layer are decreased for higher Prandtl number Pr. From Fig 18 it is clearly examined that a weaker concentration profile *ϕ*(*ζ*) is generated by higher Brownian motion parameter *N*_*b*_. Fig 19 shows that the larger thermophoresis parameter *N*_*t*_ yields a higher concentration profile *ϕ*(*ζ*). Table 2 is calculated for numerical computations of local Nusselt number $Re_{x}^{-1/2}Nu_{x}$ via *β*_1,_ *λ*_1_, *Ha*, *γ*, *S*_1,_ *N*_*t*_, *N*_*b*,_ *Le* and Pr when *n* = 1.5. Here we noticed that the local Nusselt number has higher values for larger Prandtl number Pr while opposite trend is noticed for Lewis number *Le*. It is also observed that *λ*_1_, *S*_1_ and *Ha* yield lower local Nusselt number. The local Deborah number *β*_1_ shows opposite behavior for local Nusselt number when compared with aforementioned parameters.

**Table 2:** Numerical calculations of local Nusselt number $\text{R}{\text{e}}_{x}^{-1/2}Nu_{x}$ for different values of *β*1, *λ*_1,_ *Ha*, *γ*, *S*_1,_ *N*_*t*_, *N*_*b*,_ *Le* and Pr when *n* = 1.5.

*β*_1_	*λ*_1_	*Ha*	*γ*	*S*_1_	*N*_*t*_	*N*_*b*_	*Le*	Pr	$Re_{x}^{-1/2}Nu_{x}$
0.0	0.3	0.2	0.3	0.1	0.3	0.5	1.0	1.2	0.2060
0.3									0.2158
0.6									0.2210
0.2	0.0	0.2	0.3	0.1	0.3	0.5	1.0	1.2	0.2187
	0.5								0.2098
	1.0								0.2004
0.2	0.3	0.2	0.3	0.1	0.3	0.5	1.0	1.2	0.2133
		0.5							0.2088
		0.8							0.2003
0.2	0.3	0.2	0.1	0.1	0.3	0.5	1.0	1.2	0.0941
			0.6						0.3107
			1.2						0.4007
0.2	0.3	0.2	0.3	0.0	0.3	0.5	1.0	1.2	0.2282
				0.1					0.2133
				0.2					0.1839
0.2	0.3	0.2	0.3	0.1	0.0	0.5	1.0	1.2	0.2151
					0.5				0.2119
					1.0				0.2082
0.2	0.3	0.2	0.3	0.1	0.3	0.5		1.2	0.2133
						1.0	1.0		0.2133
						1.5			0.2133
0.2	0.3	0.2	0.3	0.1	0.3	0.5	0.5	1.2	0.2140
							1.0		0.2133
							1.5		0.2128
0.2	0.3	0.2	0.3	0.1	0.3	0.5	1.0	1.0	0.2017
								1.5	0.2260
								2.0	0.2403

## 6. Conclusions

Magnetohydrodynamic (MHD) flow of Jeffrey nanofluid bounded by a nonlinear stretching surface with heat generation/absorption is investigated. The observations are summarized in the following points.

- An increase in local Deborah number *β*_1_ depicts a decreasing behavior for temperature *θ*(*ζ*) and concentration *ϕ*(*ζ*) profiles.
- Both temperature *θ*(*ζ*) and concentration *ϕ*(*ζ*) profiles are enhanced when ratio of relaxation to retardation times *λ*_1_ is increased.
- An increase in Hartman number *Ha* shows higher temperature *θ*(*ζ*) and concentration *ϕ*(*ζ*) profiles.
- Biot number *γ* has similar effects for temperature *θ*(*ζ*) and concentration *ϕ*(*ζ*) profiles.
- Prandtl number Pr indicates qualitatively similar behavior for both temperature *θ*(*ζ*) and concentration *ϕ*(*ζ*) profiles.
- Temperature profile *θ*(*ζ*) and associated thermal layer thickness are increasing functions of heat generation/absorption parameter *S*_1_.
- Concentration profile *ϕ*(*ζ*) decays for larger Brownian motion parameter *N*_*b*_.
- Increasing behavior is noted for temperature *θ*(*ζ*) and concentration *ϕ*(*ζ*) profiles for larger thermophoresis parameter *N*_*t*_.
- Local Nusselt number reduces for larger *N*_*t*_ but it remains constant for *N*_*b*_.
